# Supplementary material for: Learning curve and surgical outcome of robotic assisted colorectal surgery with ERAS program
Source: Sci Rep. 2022 Nov 29;12:20566. doi: 10.1038/s41598-022-24665-w (PMC9709162; doi:10.1038/s41598-022-24665-w)
Supplement: Supplementary file 2 — Supplementary Information 2. [file 41598_2022_24665_MOESM2_ESM.zip › RAL Raw data-Ñ[▒K/080/OP N_ñw╝╨░O▒Kñσ.pdf]

W55 / W55

臺中榮民總醫院
手術室護理記錄

[redacted]

基本資料

- 室別： 開刀房-17室，手術科別： CRS，手術類別： 常規，日期： 2021/05/06
- 進入時間： 1250 ； 麻醉開始時間： 1300 ； Time Out時間： 1435 ； 劃刀時間： 1436 ； 手術結束時間： 1930 送出時間： 1950 ；
- 麻醉方式： GE
  診斷： Malignant tumor of rectum
  手術名稱： Robot Assisted Low Anterior Resection
  手術局部用藥： nil
  術前預防性抗生素：
  - 抗生素名稱一： Veterin 1GM IVD/由麻醉護理師給予
  - 時間： 1415
  - 上次簽章: 呂昭蓉

  ，追加預防性抗生素：
- 手術醫師： 蔣鋒帆，林陳彥
- 麻醉醫師： VS 陳永銘
-
  - 刷手護士A ： 林怡廷，時間： 1250，刷手護士B ： 陳姸希，時間： 1250，接替刷手一： 張秀敏，時間： 1500，接替刷手二： 林怡廷，時間： 1630
  - 流動護士A ： 呂昭蓉，時間： 1250，接替流動一： 陳盈妤，時間： 1500，接替流動二： 張秀敏，時間： 1700
- 更多接替及流動護士： 接替刷手三： 邱欐嫊，時間： 1800，接替流動三： 吳貞儀，時間： 1800

皮膚狀況

A-V Shunt： 無
術前： 完整
記錄者： 上次簽章: 呂昭蓉
術後： 完整
記錄者： 上次簽章: 吳貞儀

手術準備

手術臥式 單一手術臥式

-
  - 手術臥式： 截石臥式
  - 使用附件： 溫毯 38 ℃，頭圈，腳架
  - 皮膚準備： 10%Aq Betadine，Hibiscrub，2% Chlorhexidine
  - 電燒： 使用電燒: 電極片位置： 右大腿，記錄者： 上次簽章: 呂昭蓉

止血帶T： 無

植入物

- 植入物名稱/號碼/數量/部位： GIA45-4.3*2/RECTUM

，記錄者： 上次簽章: 張秀敏

標本送檢

- 細菌培養 無
- 細胞學 無
- 冰凍切片 無
- 病理標本
  組織名稱一： IMA root
  ，
  組織名稱二： distal cut end
  ，
  組織名稱三： rectum

- 截肢 無

標本交付 標本： 2 袋 ， 標本： 0 盒 ， 標本： 1 瓶 ， 細胞學： 0 管 ， Culture Tube： 0 管 ， 被交付醫師： CR 林陳彥 ，

- 記錄者： 上次簽章: 吳貞儀
- 時間： 1845

傷口術後敷料

傷口敷料： 傷口敷料： 無

- 請於圖上標示部位：

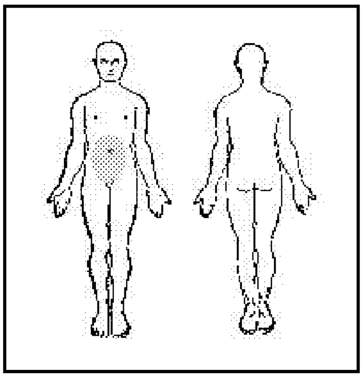

- 傷口敷料： 軟性敷料

導管

導管	天數	導管資訊	插入日期	插入時間	更換日期	拔除日期	拔除時間	拔除原因	預計拔除/更換	預計拔除/更換日期
Epidural PCA(1)	1	部位:硬脊膜;	2021/05/06	1330						
Double Lumen CVC(1)	1	號碼:8 Fr.; 深度:15cm; 部位:jugular (右); 象限:RU;	2021/05/06	1350						
Sump NG Tube(1)	1	號碼:16 Fr.; 部位:胃; 註記:58cm;	2021/05/06	1400						
Foley(1)	1	號碼:16 Fr.; 部位:膀胱;	2021/05/06	1335						
A-line (1)	1	號碼:20 G; 部位:左手;	2021/05/06	1340						

用物計數

- 手術中出血量： minimal c.c.
- 手術中尿量： 1050 c.c.
-
  - 其它： 無

用物計數： 多切口同時縫合

- 1.器械： 正確 ，2.縫針： 正確 ，3.刀片： 正確 ，4.紗布： 正確 ，5.腹部墊： 未使用 ，6.紗球： 未使用 ，7.PEANUTS ： 未使用 ，8.紗條： 未使用 ，9.絲帶： 未使用 ，10.針頭： 未使用 ，11.Vessel loop： 未使用 ，12.Boots： 未使用 ，13.Jelco： 未使用 ，14.棉條： 未使用 ，
  - 第一次：
    - 刷手護士 ： 邱欐婇
    - 流動護士： 上次簽章: 張秀敏
    - 時間： 1815

  ,
  - 第二次：
    - 刷手護士 ： 邱欐婇
    - 流動護士： 上次簽章: 張秀敏
    - 時間： 1820

  ,
  - 第三次：
    - 刷手護士 ： 邱欐婇
    - 流動護士： 上次簽章: 張秀敏

- 時間： 1825

備註

備註： Dsvinci 30度scope NO.1使用
Ｒ６腹腔鏡ＮＯ．２

- 記錄者： 上次簽章: 張秀敏
- 時間： 1250

填寫護理人員 吳貞儀

正式摘要

W55 / W55

doctorRank:B
